# Macrophage colony-stimulating factor (M-CSF) is an intermediate in the process of luteinizing hormone-induced decrease in natriuretic peptide receptor 2 (NPR2) and resumption of oocyte meiosis

**DOI:** 10.1186/s13048-017-0364-x

**Published:** 2017-10-04

**Authors:** Wenchao Sun, Chang Liu, Ying Feng, Guangchao Zhuo, Wenjing Zhou, Xiaoyang Fei, Zhifen Zhang

**Affiliations:** 1 Center of Reductive Medicine, Hangzhou Obstetrics and Gynecology Hospital, Nanjing Medical University, Hangzhou, China; 2 Department of Gynecology, Hangzhou First People’s Hospital, Nanjing Medical University, Hangzhou, China; 3 Division of Embryo Laboratory, Center of Reductive Medicine, Hangzhou Obstetrics and Gynecology Hospital, Nanjing Medical University, Hangzhou, China; 4 Central Laboratory, Hangzhou First People’s Hospital, Nanjing Medical University, Hangzhou, China; 5 Department of Gynecological Endocrinology, Hangzhou Obstetrics and Gynecology Hospital, Nanjing Medical University, 369 Kunpeng Road, Hangzhou, 310008 China

**Keywords:** Luteinizing hormone, Macrophage colony-stimulating factor, Meiosis resumption, Natriuretic peptide receptor 2, Oocyte

## Abstract

**Background:**

Luteinizing hormone (LH) regulation of the ligand, natriuretic peptide precursor type C, and its receptor, natriuretic peptide receptor 2 (NPR2), is critical for oocyte maturation; however, the mechanism is not fully understood. Macrophage colony-stimulating factor (M-CSF) has recently been shown to be involved in oocyte maturation and ovulation. In the present study we determined whether or not M-CSF plays a role in the intermediate signal that mediates LH regulation of NPR2 in resumption of oocyte meiosis.

**Methods:**

Immature female C57BL/6 mice were injected i.p. with 5 IU of equine chorionic gonadotropin (eCG) to stimulate follicle development. After 44–48 h, the eCG-stimulated mice were injected i.p. with an ovulatory dose of 5 IU of human chorionic gonadotropin (hCG). The ovaries were excised at selected times. Pre-ovulatory follicles (POFs) and cumulus-oocyte complexes were cultured in different media. Immunohistochemical and quantitative real-time PCR analyses were used to assess the expression of M-CSF, M-CSF receptor (M-CSF-R), and NPR2. The presence of germinal vesicle breakdown (GVBD) was examined under a stereomicroscope to morphologically evaluate resumption of oocyte meiosis.

**Results:**

NPR2 was mainly expressed in cumulus cells of pre-ovulatory follicles, while M-CSF and M-CSF-R were expressed in both mural granulosa and cumulus cells. The levels of M-CSF/M-CSF-R and NPR2 decreased within 4 h after treatment of hCG. M-CSF not only reduced the expression of NPR2 mRNA via its receptor (M-CSF-R), but also increased the proportion of GVBD in oocytes.

**Conclusion:**

M-CSF serves as an intermediate signal, thus inducing a vital decrease in the NPR2 levels in cumulus cells, and regulates the process of LH-induced resumption of meiosis.

## Background

**Fig. 1:**
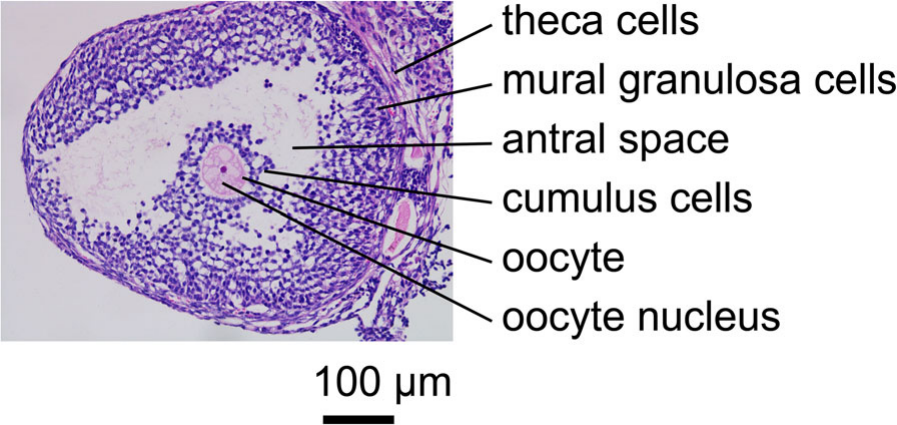
Histologic section of a murine ovary, showing structures and cell types in the follicle

In female mammals, oocytes grow and undergo meiosis over a prolonged period of time [1, 2]. Once the growing follicles reach the early antral stage, oocytes acquire meiotic competence [3]; however, oocytes are arrested at the diplotene stage of the first meiotic prophase because signals from the surrounding granulosa cells (GCs) prevent the machinery required for resumption of meiosis [3, 4]. Throughout prophase arrest, the oocyte is situated in a follicle where the oocyte is encircled by GCs (Fig. 1). The essence of signals maintaining meiotic arrest has been demonstrated as a complicated interaction between cyclic adenosine 3′,5′-monophosphate (cAMP) and cyclic guanosine 3′,5′-monophosphate (cGMP) signaling [5–10]. Cyclic AMP is generated by the oocyte via the activation of Gs G-protein by the G-protein-coupled receptor and adenylyl cyclases. Cyclic GMP, synthesized in surrounding GCs, diffuses into the oocyte through the network of gap junction communications, and inhibits oocyte cAMP-phosphodiesterase (PDE) 3A activity and hydrolysis of cAMP to maintain meiotic arrest [2–7, 9, 11, 12]. Subsequent studies have indicated that generation of cGMP is stimulated by a paracrine loop, which includes natriuretic peptide receptor 2 (NPR2) and the ligand, natriuretic peptide precursor type C (NPPC) [13]. NPPC, produced by mural GCs, activates NPR2, which is produced mainly by cumulus cells surrounding the oocyte, increases cAMP and cGMP levels in the oocyte, and prevents spontaneous (gonadotropin-independent) resumption of oocyte meiosis [13, 14]. In mice deficient in the ligand, NPPC, or its cognate receptor, NPR2, oocytes precociously re-enter the meiotic cell cycle as soon as the oocytes reach the early antral follicle stage [13, 15].

Oocytes resume meiosis when the luteinizing hormone (LH) surge causes dramatic changes in pre-ovulatory follicles (POFs) [16, 17]. It has been shown that LH causes a spectacular decrease in NPPC in the follicles of a wide variety of mammalian species, including mice, rats, pigs, and humans [4, 5, 13, 18, 19]. Decreased NPPC in turn reduces the amount of NPR2 and cGMP, and meiosis resumes in oocytes [4, 5]. Recently, when the kinetic curve of cytokines was further studied, NPPC was not shown to be decreased until 2 h after the LH surge, whereas the decrease in cGMP was first detected at 15–20 min [14]. NPR2 also undergoes a rapid decrease in activity within 10 min after LH exposure, when the NPPC concentration is constant [20, 21]. This phenomenon (that the receptor is motivated before ligand activation) gave rise to the hypothesis that multiple pathways mediate LH regulation of NPR2 and downstream cGMP signaling in the ovarian follicle [21]. These multiple pathways include the phosphoprotein phosphatase signaling pathway [20], which has recently been associated with ovulation process. Multiple pathways are known to include the epidermal growth factor receptor (EGFR) signaling pathway [21–26], but recent findings have shown new relationships. The exact number of these multiple pathways is unknown.

Macrophage colony-stimulating factor (M-CSF), a hemopoietic growth factor with a classic function of controlling the proliferation and differentiation of macrophages, has recently been shown to be involved in oocyte maturation and ovulation [27–30]. We have previously reported that M-CSF is implicated in follicular GC function [27], and M-CSF can modulate the generation of NPPC, which may regulate ovulation triggered by LH [31]. In the present study we determined whether or not M-CSF is included in the aforementioned “multiple pathways” that mediate LH regulation of NPR2 in ovarian follicles.

## Methods

### Animals and hormone treatments

Immature (22–25 days old) female C57BL/6 mice (Zhejiang Academy of Medical Sciences, Hangzhou, China) were injected i.p. with 5 IU of equine chorionic gonadotropin (eCG) to stimulate follicle development. After 44–48 h, the eCG-stimulated mice were injected i.p. with an ovulatory dose of human chorionic gonadotropin (hCG; 5 IU). The ovaries were excised at selected times after injection and processed for immunohistochemical analysis and quantitative real-time PCR. For cell culture of POFs and cumulus-oocyte complexes (COCs), the eCG-stimulated mice were euthanized and the ovaries were excised without hCG injection. All chemicals were purchased from Sigma-Aldrich (St. Louis, MO, USA) unless otherwise stated. All animal procedures were approved by the guidelines of the Nanjing Medical University Administrative Panel on Laboratory Animal Care.

### Culture of POFs

The POFs were dissected stereomicroscopically from the ovaries of eCG-stimulated mice, as previously described [32, 33]. The POFs were placed in minimum essential media (MEM)-α supplemented with 100 mg/ml of fetal bovine serum (FBS), 100 U/ml of penicillin G, and 100 μg/ml of streptomycin sulfate. After equilibration, follicles (10–15 per group) were cultured at 37 °C in an atmosphere of 5% O_2_, 5% CO_2_, and 90% N_2_ for the indicated time in the presence or absence of hCG (5 IU/ml). At the end of the culture period, follicles were collected for quantitative RT-PCR analysis to measure hCG regulation of NPR2 transcript levels.

### Culture of COCs

COCs were obtained by puncturing the POFs in the ovaries from eCG-stimulated mice. After isolation, COCs were washed in the medium and cultured for 2 h. The culture medium was MEM-α supplemented with 100 mg/ml of FBS, 100 U/ml of penicillin G, and 100 μg/ml of streptomycin sulfate with or without 30 nM NPPC. At least 10 COCs per treatment group were cultured. Cultures were maintained under a controlled atmosphere of 5% O_2_, 5% CO_2_, and 90% N_2_ at 37 °C. After culture, COCs were collected for quantitative RT-PCR to calculate the NPR2 transcript levels. The presence of germinal vesicle breakdown (GVBD) was examined under a stereomicroscope.

### RNA isolation, reverse transcription, and quantitative real-time PCR

Mouse ovaries, cultured follicles, and COCs were collected in 350 μl of RNeasy lysis buffer. The tissues and cells were stored at −80 °C until analysis for mRNA expression. Total RNA was isolated from frozen samples using the RNeasy micro-RNA isolation kit (Qiagen, Valencia, CA, USA), as recommended by the manufacturer’s instructions. Reverse transcription and real-time PCR was then carried out to quantify the steady-state mRNA levels of NPPC, NPR2, M-CSF, and M-CSF-R using an ABI 7500 real-time PCR instrument (Applied Biosystems, Foster City, CA, USA). The housekeeping gene, Rpl19, was considered the internal control. The primers for real-time PCR of NPPC, NPR2, M-CSF, M-CSF-R, and Rpl19 are listed in Table 1. The levels of NPPC, NPR2, M-CSF, and M-CSF-R mRNA were first normalized to the level of Rpl19 expression, then demonstrated relative to a control group in which the level of expression was set at 1. Each experiment was repeated independently at least three times.

**Table 1:** Primer sequences, forward (F) or reverse (R), used for quantitative RT-PCR

Gene	F or R	Primer sequence
NPPC	F	GGGAGCCAATCTCAAGGGAG
	R	GTTGCCGCCTTTGTATTTGC
NPR2	F	GCATTGTCACCGAGTATTGTCC
	R	CAGACCGTAATCTGTTATTTTGAGC
M-CSF	F	TGATTGGGAATGGACACCTG
	R	AAAGGCAATCTGGCATGAAGT
M-CSF-R	F	GGTGGCTGTGAAGATGCTAAAG
	R	AGGCTCCCAAGAGGTTGACTAT
Rpl19	F	CCGCTGCGGGAAAAAGAAG
	R	CAGCCCATCCTTGATCAGCTT

### Immunohistochemistry

The excised ovaries were fixed in 10% formalin. After dehydration, the fixed ovaries were embedded in paraplast, then sectioned at 5-μm intervals onto Superfrost Plus microscope slides. For immunohistochemical staining, sections of ovaries were deparaffinized and rehydrated, treated with 3% H_2_O_2_ for 20 min to inactivate intrinsic peroxidase activity, and incubated with ethylene dinitrilo tetraacetic acid buffer for 10 min for antigen retrieval. Washes were carried out with automation phosphate buffer. Sections were incubated for 2 h at 4 °C with rabbit anti-mouse antibody diluted 1:200 in buffer containing 5% bovine serum albumin. Sections were next incubated with horseradish peroxidase-labeled goat anti-rabbit antibody for 50 min at room temperature. Staining was achieved using diaminobenzidine chromogen. The staining reactions were stopped with distilled water, and sections were dehydrated and mounted with neutral balsam.

### Image analysis of densitometry

Slides were examined under a microscope with a 200 × objective. The obtained images were captured and examined by Image Pro Plus 6.0 software (Media Cybernetics, Inc., Washington, USA). The integrated optical density (IOD) was calibrated, and the area of interest was set. The mean optical density was defined as the IOD divided by the total area examined.

### Statistical analysis

Statistical analyses were carried out using SPSS software (version 16.0: SPSS, Inc., Chicago, IL, USA). Data are presented as the mean ± SEM. Differences between experimental and control groups were analyzed by ANOVA test. Statistical significance was set at *P* value of less than 0.05.

## Results

### Localization of M-CSF, M-CSF-R, and NPR2 in POFs

**Fig. 2:**
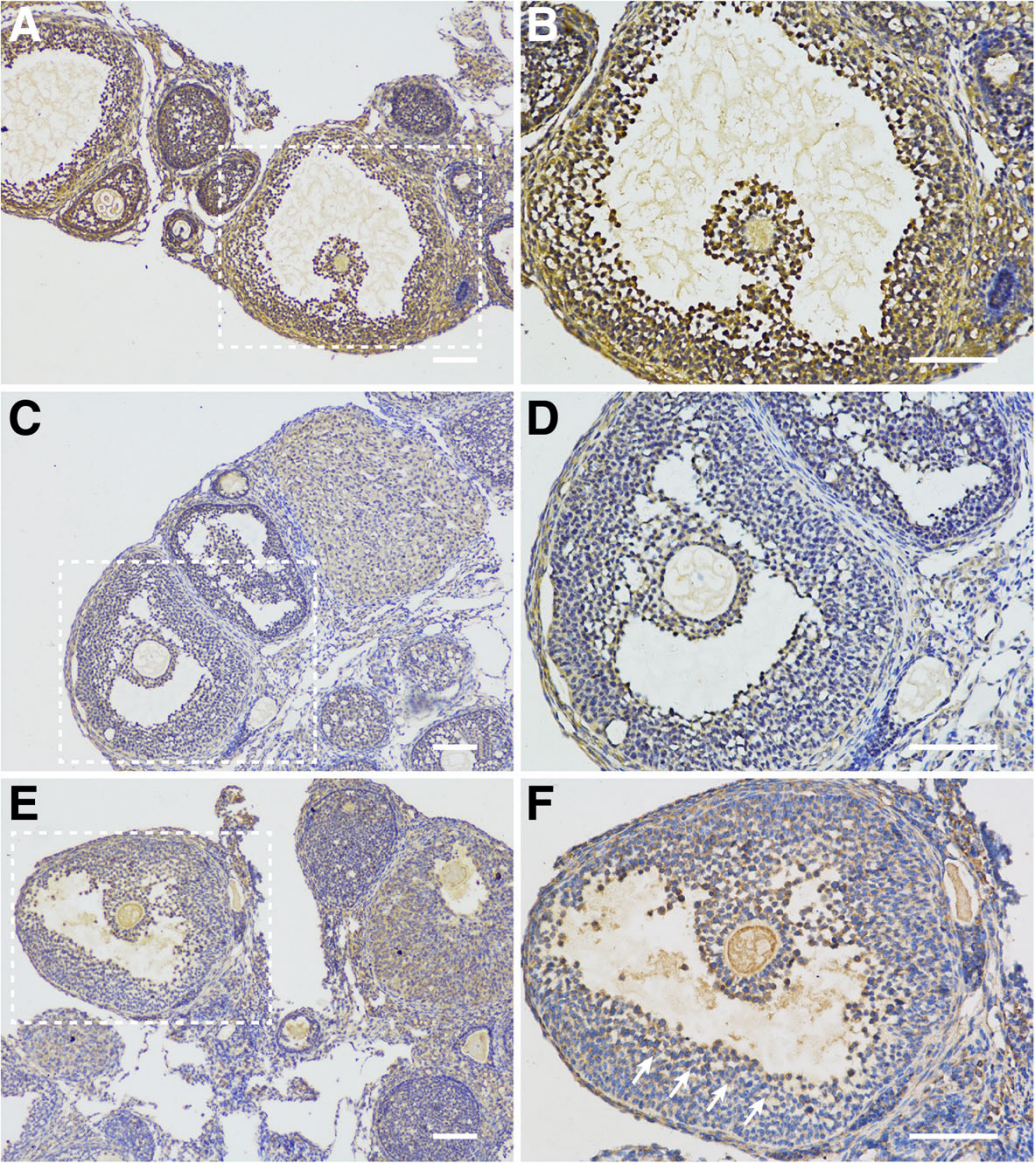
Expression patterns of M-CSF, M-CSF-R, and NPR2 in the ovaries of eCG-stimulated mice. Localization of M-CSF (**a** and **b**), M-CSF-R (**c** and **d**), and NPR2 (**e** and **f**) was analyzed using immunohistochemistry in the ovaries of eCG-stimulated mice. **b**, **d**, and **f** are enlarged views of the white boxed areas in **a**, **c**, and **e**, respectively. White arrows indicate periantral mural GCs. The results are representative of three ovaries for each experiment. Scale bar, 100 μm

To investigate if M-CSF, M-CSF-R, and NPR2 signaling is involved in regulation of oocyte meiosis in POFs. We analyzed M-CSF, M-CSF-R, and NPR2 localization in ovarian sections from eCG-stimulated mice. Immunohistochemistry analysis revealed that M-CSF and M-CSF-R are expressed in both mural GCs and cumulus cells (Fig. 2a-d), while the expression of NPR2 in POFs was mainly detected in cumulus cells and was also observed in peri-antral mural GCs (GCs located on the lining of antral spaces, [Fig. 2e and f]).

### Changes in expression of M-CSF, M-CSF-R, and NPR2 in the ovaries of mice injected with gonadotropin

**Fig. 3:**
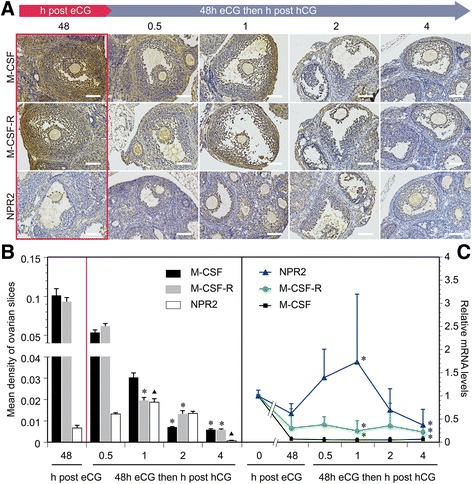
Gonadotropin control of M-CSF, M-CSF-R and NPR2 expression in ovaries in vivo. **a** Localization of M-CSF, M-CSF-R, and NPR2 in ovarian follicles shown with immunohistochemistry. Red box represents the time point 48 h after eCG treatment. Scale bars, 100 μm. **b** Mean optical density of ovarian slides. *, *P* < .05; ▲, *P* < .01 compared with corresponding 48-h value in the red box. Bars show the mean ± SEM of three independent slides. **c** Expression of M-CSF, M-CSF-R, and NPR2 mRNA by quantitative RT-PCR. The value in the control (0-h eCG) was set at a value of 1, and levels of expression in other samples are demonstrated relative to the control. *, *P* < .05 compared with the corresponding control. Bars show the mean ± SEM of three independent experiments

We further elucidated the importance of M-CSF, M-CSF-R, and NPR2 signaling in LH-induced resumption of oocyte meiosis. Using immunohistochemical techniques, we determined the changes in expression of M-CSF, M-CSF-R, and NPR2 in the ovaries of eCG-stimulated mice at 0 (48 h after eCG treatment), 0.5, 1, 2, and 4 h after injection with hCG (Fig. 3a). The mean optical density was calculated and showed that M-CSF and M-CSF-R expression was gradually decreased within 4 h after hCG treatment, which was administered 48 h after eCG injection (Fig. 3b). NPR2 expression peaked at 1 h, but an obvious reduction in expression was detected at 4 h (Fig. 3b). Expression of M-CSF, M-CSF-R, and NPR2 mRNA was also detected (Fig. 3c) and was consistent with the results of immunohistochemistry and densitometry analysis.

### Kinetic curve of NPR2 mRNA levels controlled by hCG in vitro

**Fig. 4:**
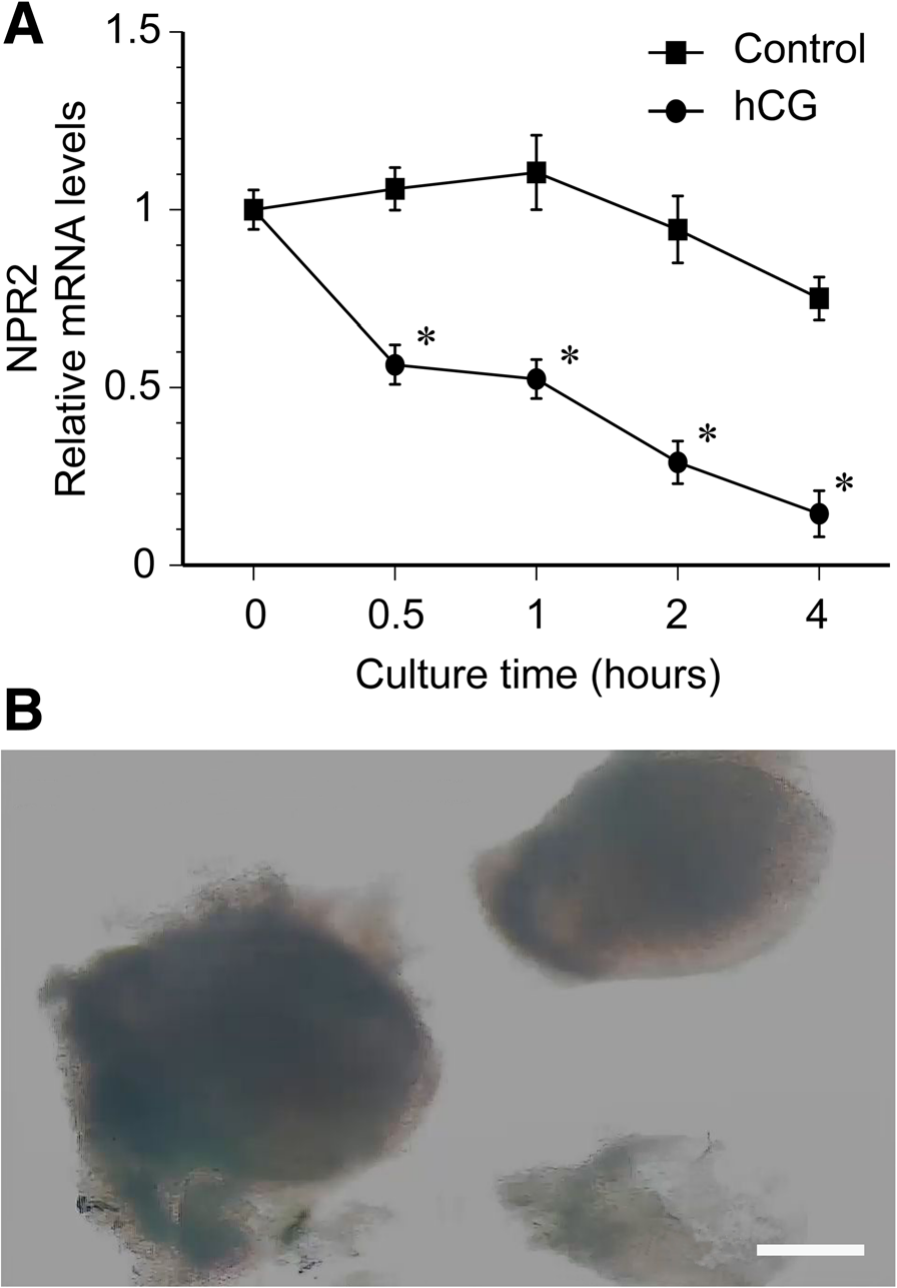
Effect of hCG on NPR2 mRNA expression in POFs. **a** The kinetics of hCG-induced NPR2 mRNA levels in POFs. *, *P* < .05 compared with corresponding control. Bars demonstrate the mean ± SEM of three independent experiments with at least 10 POFs evaluated at each group in each experiment. **b** The image of cultured POFs. Scale bar, 100 μm

To study the effect of hCG on the kinetic curve of NPR2 mRNA levels in POFs, we examined the expression of NPR2 mRNA after 0, 0.5, 1, 2, and 4 h of culture after hCG treatment. The hCG (5 IU/ml) significantly decreased NPR2 mRNA levels by one-half at 0.5 h, and nearly 90% at 4 h of culture. In the control group without hCG treatment, the levels of NPR2 mRNA in POFs were slightly increased after POFs were cultured for 1 h. Then, the NPR2 mRNA levels were only slightly decreased after POFs were cultured for 4 h (Fig. 4a). The image of cultured POFs is presented in Fig. 4b. The results are in agreement with the speculation that hCG regulates NPR2.

### M-CSF/M-CSF-R signaling decreases the level of NPR2 mRNA in cumulus cells and contributes to resumption of meiosis in oocytes

**Fig. 5:**
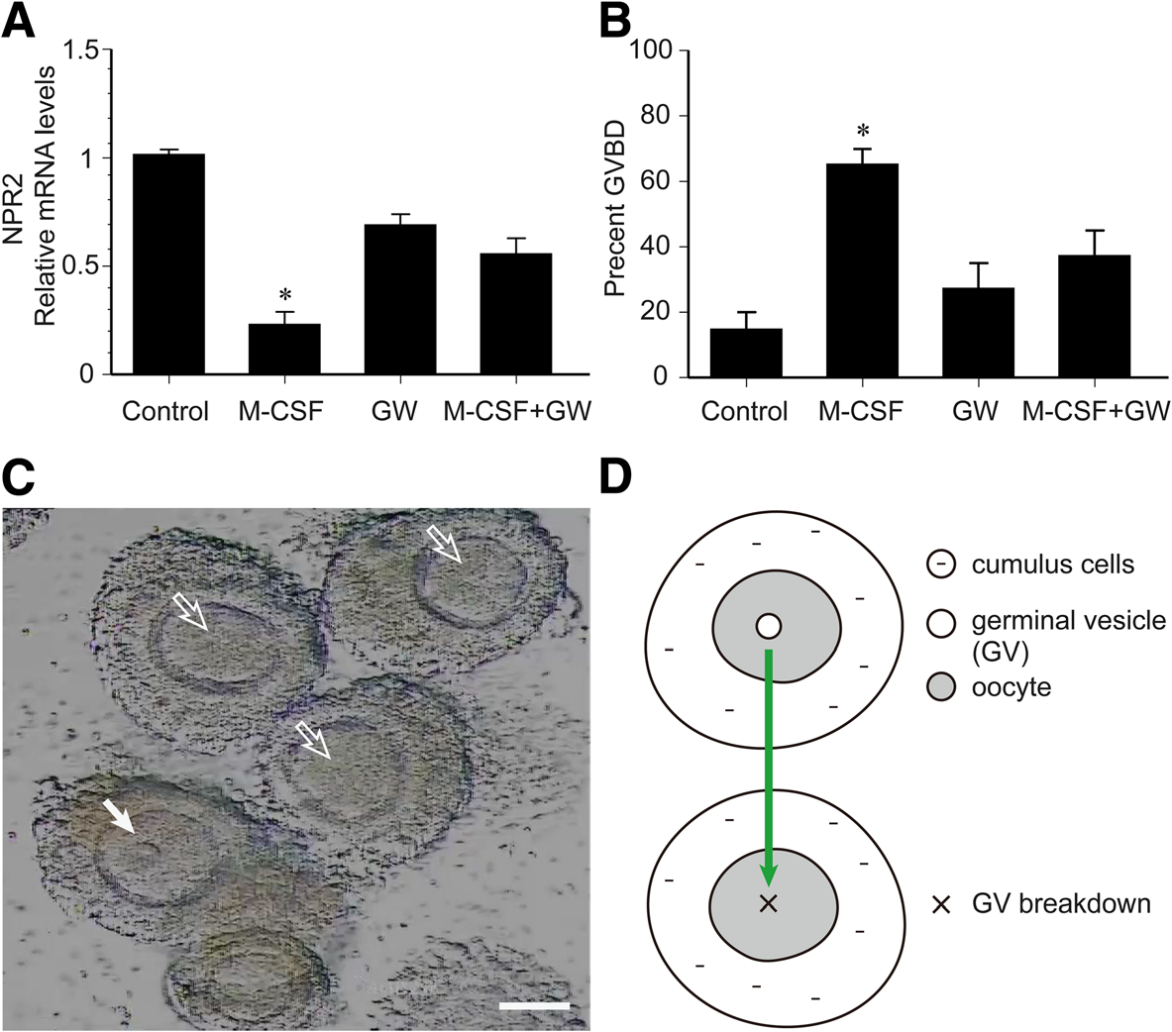
Effect of M-CSF on NPR2 mRNA expression and oocyte maturation. COCs isolated from eCG-stimulated mice were cultured in MEM-α in the presence of 30 nM NPPC (control), to which was added 200 ng/ml M-CSF and/or 1 μmol/L GW2580 for 2 h. **a** Effect of GW2580 on M-CSF-induced NPR2 mRNA levels in cumulus cells after 2 h of culture. *, *P* < 0.05 compared with control. **b** Effect of GW2580 on M-CSF-induced resumption of oocyte meiosis after 2 h of culture. *, *P* < 0.05 compared with control. Bars demonstrate the mean ± SEM of three independent experiments with at least 10 COCs evaluated at each group in each experiment. **c** Presence of GVBD (hollow arrows) or intact germinal vesicle (GV, solid arrows) in oocytes at 2 h of COC culture. Scale bar, 25 μm. **d** Onset of meiotic resumption in oocytes (green arrows). GW, GW2580

The effect of M-CSF/M-CSF-R signaling on NPR2 mRNA expression and oocyte maturation was determined using cultured COCs isolated from POFs of eCG-stimulated mice. Isolated COCs spontaneously resume meiosis because mural GCs containing the inhibitory molecule, NPPC, is removed. Therefore, a culture system supplemented with 30 nM NPPC was adopted to maintain meiotic arrest in oocytes [23]. M-CSF (200 ng/ml) significantly decreased the level of NPR2 mRNA when COCs were cultured for 2 h (Fig. 5a). M-CSF stimulated GVBD in approximately 65% of oocytes at 2 h of culture (Fig. 5b and c). GVBD marks the onset of meiotic resumption and is the key event in oocyte maturation (Fig. 5d). Furthermore, the M-CSF-induced decrease in NPR2 and resumption of oocyte meiosis was partially inhibited by GW2580 (a selective M-CSF-R inhibitor purchased from Selleckchem, Houston, TX, USA; Fig. 5a and b), suggesting that M-CSF functioned via the activity of the M-CSF receptor.

## Discussion

In the present study, we focused on the effect of M-CSF in the regulation of oocyte meiosis, and identified some crucial roles, including: (a) NPR2 was mainly expressed in cumulus cells of POFs, while M-CSF and M-CSF-R were expressed in both mural GCs and cumulus cells; (b) the levels of M-CSF/M-CSF-R and NPR2 decreased within 4 h after hCG treatment; and (c) M-CSF not only reduced the expression of NPR2 mRNA via its receptor (M-CSF-R), but also increased the proportion of GVBD of oocytes, which indicates that M-CSF is an intermediate signal, inducing a vital decrease in NPR2 levels in cumulus cells, and regulates the process of LH-induced resumption of meiosis.

M-CSF extensively participates in the processes of ovulation [27, 29]. Female mice lacking the coding region for the M-CSF gene (M-CSF deficient) have remarkably lower ovulation rates compared to the wild-type counterparts. Indeed, administration of M-CSF from birth to reinstate circulating M-CSF levels could reverse these defects [27]. In humans, high serum concentrations of M-CSF were related to successful oocyte retrieval during in-vitro fertilization and embryo transfer cycles [28]. LH surge triggers dramatic changes in cytokines during ovulation [16]. Although the changes in M-CSF and M-CSF-R 24 h after a LH surge are clear [29], the alteration in M-CSF/M-CSF-R within a short period of time (4 h) following the LH surge is unknown. Therefore, we studied the changes in M-CSF/M-CSF-R expression in the ovaries of mice injected with gonadotropin. The data from our study showed that M-CSF/M-CSF-R, expressed in both mural GCs and cumulus cells, was gradually decreased within 4 h after hCG treatment. We hypothesized that this change is related to estradiol (E_2_). Reportedly, the expression of M-CSF was enhanced by E_2_ in luteinized GCs in a dose-dependent manner in vitro [27]. E_2_ maintains cumulus cell expression of NPR2 and inhibits the resumption of meiosis in mouse oocytes in vitro [34], and the level decreased during ovulation. Thus, decreased E_2_ has a certain role to the resumption of oocyte meiosis and ovulation. Therefore, the decreased level of M-CSF after hCG treatment may be due to the decreased levels of E_2_. Although the hypothesis is theoretically feasible, it is still necessary to further investigate whether or not a decrease in M-CSF during ovulation is related to a decrease in the E_2_ level.

Recent studies have revealed that cGMP stimulated by NPR2 from cumulus cells diffuses into oocytes via gap junctions and controls cAMP concentration through inhibition of PDE3A activity, indicating that higher NPR2 levels in cumulus cells is responsible for oocyte meiotic arrest by maintaining high cAMP levels in oocytes [1, 4–7]. Our results showed that NPR2 is primarily expressed in cumulus cells surrounding oocytes, which is consistent with the literature. The results presented herein support the thought that control of NPR2, which is expressed in cumulus cells, is essential for maintaining meiotic arrest in pre-ovulatory oocytes [4, 13]. LH reduces the activity of NPR2 during ovulation, and promotes resumption of meiosis in oocytes [14]. In particular, our findings demonstrated that NPR2 is also expressed in peri-antral mural GCs (GCs situated on the antral side), which substantiates the viewpoint that some mGCs are activated by NPPC in an autocrine process to raise cGMP levels [24, 34]. NPR2 was down-regulated following a specific time curve after hCG injection. The kinetic curve of NPR2 after hCG treatment in our study was similar to that reported in the literature [23], but there were some differences. In our research, NPR2 expression peaked 1 h after hCG treatment, and an obvious reduction of approximately 85% in expression was detected at 4 h in vivo. The rise in NPR2 expression within 2 h after LH treatment was different from that reported in the literature, likely due to the long half-life (40 ~ 120 h) of eCG administered 48 h before hCG injection to stimulate follicle development [22, 24]. The eCG promoted the up-regulation of NPR2 during follicle growth [33]. To clarify the NPR2 expression change within 2 h after LH treatment, cultured POFs were used to examine the kinetic curve of NPR2 mRNA levels controlled by hCG in vitro. The data from our study were consistent with the literature [23]. We did not observe a significant increase in expression of NPR2 in hCG-containing media in vitro, possibly because in vitro culture eliminated the residual effect of eCG. To further declare the effect of M-CSF/M-CSF-R signaling on NPR2 mRNA expression and oocyte maturation, COCs isolated from POFs were cultured. The results showed that the M-CSF/M-CSF-R signaling reduced levels of NPR2 mRNA in cumulus cells and promoted resumption of oocyte meiosis. Then, we added GW2580 to restrict the effect of M-CSF signaling in vitro. The results showed that the effect of M-CSF at 4 h was partially abolished.

## Conclusion

We conclude that M-CSF is an intermediate signal, inducing a vital decrease in NPR2 levels in cumulus cells, and regulates the process of LH-induced resumption of meiosis. Although further research is needed, our findings bring forth powerful evidence to interpret “multiple pathways” that mediate LH regulation of NPR2 in the process of resuming oocyte meiosis.

## Abbreviations

cAMP: cyclic adenosine 3′,5′-monophosphate
cGMP: cyclic guanosine 3′,5′-monophosphate
COCs: Cumulus-oocyte complexes
E_2_: Estradiol
eCG: Equine chorionic gonadotropin
EGFR: Epidermal growth factor receptor
FBS: Fetal bovine serum
GCs: Granulosa cells
GVBD: Germinal vesicle breakdown
hCG: Human chorionic gonadotropin
IOD: Integrated optical density
LH: Luteinizing hormone
M-CSF: Macrophage colony-stimulating factor
M-CSF-R: M-CSF receptor
MEM: Minimum essential media
NPPC: Natriuretic peptide precursor type C
NPR2: Natriuretic peptide receptor 2
PDE: Phosphodiesterase
POFs: Pre-ovulatory follicles
